# Atopic disease and astigmatism: a population-based study

**DOI:** 10.1038/s41433-025-03712-6

**Published:** 2025-02-28

**Authors:** Margarita Safir, Itay Nitzan, Yair Hanina, Ari Safir, Eliya Levinger, Dan Heller, Nir Sorkin

**Affiliations:** 1 Ophthalmology Department, Rabin Medical Center, Faculty of Medicine, Tel Aviv University, Tel Aviv, Israel; 2 Department of Military Medicine, Faculty of Medicine, The Hebrew University of Jerusalem, Jerusalem, Israel; 3 Department of Ophthalmology, Hadassah-Hebrew University Medical Center, Jerusalem, Israel; 4 Department of Dermatology, Tel Aviv Medical Center, Faculty of Medicine, Tel Aviv University, Tel Aviv, Israel; 5 Department of Ophthalmology, Tel Aviv Medical Center, Faculty of Medicine, Tel Aviv University, Tel Aviv, Israel

**Keywords:** Risk factors, Epidemiology

## Abstract

**Objectives:**

To assess the relationship between atopic disease and astigmatism in adolescence and young adulthood.

**Methods:**

In this population-based cross-sectional study 897,811 medical records of Israeli adolescents and young adults without keratoconus were reviewed. The prevalence of low-to-moderate (3.00 > D ≥ 0.75) and high ( ≥ 3.00 D) astigmatism were calculated in cases with and without atopic disease, including ocular atopic disease (OAD), asthma, allergic rhinitis, atopic dermatitis, angioedema/urticaria, and history of anaphylaxis. Relationships were analysed using multinominal logistic regression, with adjustments for relevant sociodemographic factors.

**Results:**

A total of 897,811 adolescents were included in the analysis (mean age 17.2 ± 0.8 years, 57.8% men). OAD was found in 4702 individuals, with a prevalence of 0.5%. Adolescents with OAD demonstrated a gradual increase in odds ratio (OR) for low-to-moderate and for high astigmatism (OR 1.16, 95% CI 1.07–1.27 and OR 2.10, 95% CI 1.63–2.70, respectively). This group also showed increased OR for with-the-rule astigmatism (OR 1.34, 95% CI 1.21–1.48). Other atopic diseases were associated with more modest ORs for low-to-moderate (OR 1.09, 95% CI 1.07–1.11) and for high astigmatism (OR 1.10, 95% CI 1.02–1.19), persisting across all axis orientations. Sensitivity analysis revealed a dose-response relationship between OAD severity and astigmatism, and consistent point estimates in a group of 1331 adolescents diagnosed with OAD during military service.

**Conclusions:**

This study establishes an association between OAD and astigmatism, highlighting the importance of effective OAD management. Further research into tailored therapeutic interventions that address both conditions concurrently is needed.

## Introduction

The term ocular atopic disease (OAD) encompasses immune conjunctival disorders including atopic keratoconjunctivitis (AKC) and vernal keratoconjunctivitis (VKC), affecting up to 10.1% of the general population [1], with different prevalence according to age [2, 3]. A recent large-scale study reported its prevalence to be 0.3%, 6.6%, 18.3%, 15.8%, 8.1%, and 4.9% in infancy (<1 years), toddlerhood (1–2 years), early childhood (3–5 years), middle childhood (6–11 years), early adolescence (12–18 years) and late adolescence (18–21 years), respectively [3]. The characteristic papillary reaction on the tarsal surface, along with repetitive eye rubbing due to the ocular discomfort, lead to persistent mechanical chafing against the corneal surface in affected patients.

Keratoconus is a bilateral corneal ectasia, consisting of progressive corneal thinning, steepening, and significant astigmatism formation [4]. OAD has been demonstrated to increase the risk for keratoconus formation, presumably due to the aforementioned constant mechanical chafing [4–7]. Regardless of keratoconus diagnosis, examination of corneal biomechanics in patients diagnosed with OAD exhibits significantly higher rates of corneal irregularity compared with non-affected patients [8]. Astigmatism is a common refractive error, manifesting in 9.0–31.5% of the population [9, 10]. Astigmatism has been shown to significantly impact patients’ quality of life, visual function and educational performance, while also posing a considerable economic burden [11], particularly when exceeding the clinically significant astigmatism threshold above 1.00 dioptres [12]. Previous studies have demonstrated that repetitive eye rubbing induces reversible corneal astigmatism, which with persistent chafing may become constant or progress to true keratoconus [5, 13–17]. These findings may indicate that there is a spectrum of mechanically induced corneal distortion, where regular spectacle-corrected astigmatism is the mildest manifestation of this process. A previous study by Yangho Kin et al. examined the association between astigmatism and atopic conjunctivitis in the paediatric population attending schools in urban versus suburban areas of Korea [18]. This study reported an association between astigmatism and atopic conjunctivitis in the urban group only, which the authors attributed to higher exposure to air pollution in this group. Additional atopic disorders such as atopic dermatitis, asthma, urticaria, and anaphylaxis have also been linked to an increased risk for keratoconus diagnosis [1], but have not been evaluated in the context of astigmatism formation. Therefore, the current study aimed to evaluate the association between various atopic disorders and astigmatism.

## Methods

### Ethics

This study was approved by the Institutional Review Board of the IDF Medical Corps and adhered to the tenets of the Declaration of Helsinki.

### Patient evaluation

Israeli military pre-conscription assessment consists of standard medical evaluation and fitness-for-service (FFS) classification, taking place at around 17 years of age. This standard evaluation includes questionnaires filled by the applicant and their family physician, as well as visual acuity and refractive status data filled out by an ophthalmologist. Applicants with astigmatism of 2.00 dioptres or more are requested to undergo additional screening including corneal topography and follow-up by an ophthalmologist to rule out corneal ectasia. After all necessary candidate data are collected, one or more FFS classification numerical codes are assigned to the candidate according to their medical status. These codes are indicative of both the medical diagnosis (or similar diagnoses grouped by pathogenesis) and its severity.

Mandatory military service applies to both genders, with two to three years duration depending on the assigned military role. Therefore, follow-up data were available for all included adolescents, except for a minority who were exempted from service due to various medical and non-medical reasons. During military service, the medical status of an individual may change, with the diagnosis of new medical conditions, or by worsening/improvement of a preexisting medical condition. In such cases, a new FFS numerical code is assigned to the patient. Thus, at any given point, the individual’s FFS codes represent their most up-to-date medical status.

### Atopic disease diagnosis and severity grading

Atopic disease diagnoses were categorized into two distinct classifications: OAD and other atopic diseases. The FFS code for OAD was assigned to patients diagnosed with either AKC or VKC. For OAD FFS code assignment an ophthalmologic evaluation was required, with documentation of the duration and severity of signs and symptoms, as well as the patient’s dependence on treatment for disease control. Based on this information, a 2-point severity score was assigned. Patients who experienced persistent signs for over 3 months despite treatment, or required prolonged or permanent topical treatment, were categorized as having a moderate-severe disease. The rest were classified as having a mild disease.

For the other atopic diseases, including asthma, allergic rhinitis, atopic dermatitis, chronic angioedema/urticaria, and history of anaphylaxis FFS codes were assigned following detailed evaluation by the appropriate medical expert (pulmonologist, allergist, and dermatologist, respectively). In general, disease severity was determined by the frequency of disease exacerbations, the need for persistent medical treatment, and the presence of complications. For the purpose of analysis atopic diseases were divided into two subgroups: OAD (including AKC and VKC) and other systemic atopic disorders which do not involve the eyes directly (atopic dermatitis, allergic rhinitis, asthma, chronic angioedema or urticaria, anaphylaxis).

### Data gathering and patient selection

**Fig. 1:**
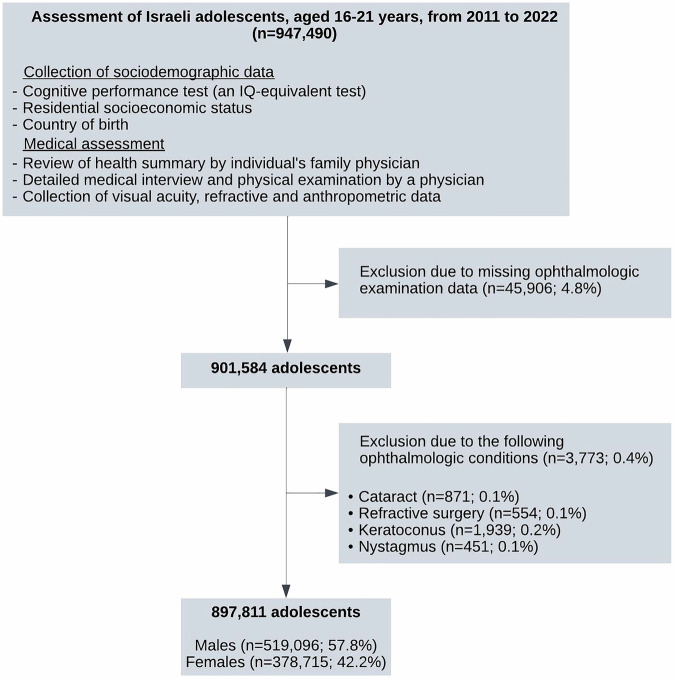
Study population selection and screening process. Medical records of all adolescents who were examined for and/or served in the IDF between 2011 and 2022 were reviewed. Cases with incomplete ophthalmological examination data, or with ophthalmological conditions potentially affecting refractive measurements were excluded.

We reviewed the medical records of all adolescents who were examined for and/or served in the IDF between 2011 and 2022. Data collected in this study included demographic characteristics (including age at FFS assignment, gender), medical FFS codes and their assignment dates. Cases with incomplete ophthalmological examination data, or with ophthalmological conditions potentially affecting refractive measurements were excluded (Fig. 1). Cases with keratoconus diagnosis throughout their military service were excluded as well.

### Statistical analysis

The entire population available was included in the analysis. Statistical analysis was performed using SPSS version 29.0 (IBM Corp., Armonk, NY, USA) and R version 4.0.2 (R Core Team, Vienna, Austria) with the package of forester. Astigmatism magnitude ranging from 0.75 up to 3.00 D was defined as low-to-moderate astigmatism, and astigmatism magnitude of 3.00D and higher was defined as high astigmatism. The chi-square test was used to compare categorical variables. We used multinomial logistic regression models to analyse the association between atopic diseases and astigmatism. We performed two main models: Model 1—analysis of astigmatism power, and Model 2—analysis of astigmatism axis orientation. We applied models without adjustment, as well as models adjusted for sociodemographic variables, acknowledging their role as possible universal confounders. A two-sided *P* value < 0.05 was considered statistically significant.

The following pre-specified sensitivity analyses were performed:

1. We examined the association between OAD severity and astigmatism.
2. We stratified the analysis by myopia status. Myopia was defined as right eye spherical equivalent ≤ –0.75 D.
3. We analysed cases of new OAD diagnoses occurring during compulsory military service.

## Results

The baseline characteristics of 897,811 adolescents included in this study are presented in Table 1. The cohort predominantly consisted of men (519,096, 57.8%), with an overall mean age of 17.2 ± 0.8 years. Overall, 4702 (0.5%) adolescents were diagnosed with OAD, 50,692 (5.6%) with asthma, 54,503 (6.1%) with allergic rhinitis, 12,562 (1.4%) with atopic dermatitis, 1282 with chronic angioedema or urticaria (0.1%), and 5,880 (0.7%) with a history of systemic allergic reaction (anaphylaxis). Adolescents with OAD or other atopic disorders were more likely to be men compared to those without atopic disease (68.7% and 63.0% vs. 57.1%, respectively). Additionally, they were more likely to achieve higher cognitive performance scores (32.7% and 31.1% vs. 27.2%, respectively) and to belong to a higher socioeconomic status (25.3% and 25.5% vs. 23.4%, respectively).

**Table 1:** Baseline characteristics of the study cohort.

	Ocular atopic disease	Other atopic disorders	No atopic disease	Total
Examinees (*n*)	4702	97,750	795,359	897,811
Sex, %				
Women	31.3	37.0	42.9	42.2
Men	68.7	63.0	57.1	57.8
Mean age ± SD (years)	17.2 ± 0.7	17.3 ± 0.8	17.2 ± 0.7	17.2 ± 0.8
Mean BMI ± SD (kg/m^2^)	22.3 ± 4.1	22.9 ± 4.5	22.7 ± 4.4	22.7 ± 4.4
Country of birth, %				
Israel	90.0	90.4	89.9	90.0
Other countries	10.0	9.6	10.1	10.0
Cognitive performance, %				
Low	30.6	31.4	34.7	34.3
Medium	36.7	37.5	38.1	38.0
High	32.7	31.1	27.2	27.6
Socioeconomic staus, %				
Low	22.2	21.7	25.8	25.3
Medium	52.6	52.9	50.8	51.0
High	25.3	25.5	23.4	23.6
BMI group, %				
Underweight	13.6	11.3	11.5	11.5
Normal weight	66.7	64.0	65.9	65.7
Overweight	14.3	16.8	15.7	15.8
Obese	5.4	7.9	6.9	7.0

*BMI* body-mass index, *SD* standard deviation.

### Atopic disease and astigmatism

Table 2 illustrates the prevalence of astigmatism power and axis orientation across categories of atopic conditions (detailed disease-specific data are provided in Supplementary Table 1 and Supplementary Fig. 1). The majority of adolescents in all categories exhibited less than 0.75 D of astigmatism, i.e. no astigmatism (overall, 86.0% of the cohort). The proportion of astigmatism in adolescents with OAD was marginally higher compared to those without any atopic disease (16.2% vs. 13.8%, *p* < 0.001). Low-to-moderate astigmatism (3.00 > D ≥ 0.75) was observed in 13.2% of the total population, with a higher proportion of 14.8% in the OAD group (*p* < 0.001). High astigmatism ( ≥ 3.00 D) was the least common, comprising only 0.7% of the total population, yet it was more prevalent in the OAD group (1.5%) compared with others (*p* < 0.001). Regarding astigmatism axis orientation, with-the-rule (WTR) astigmatism was most prevalent in the OAD group (10.3%) compared with other atopic disorders (8.9%) and no atopic disease (8.1%, *p* < 0.001). Against-the-rule (ATR) and oblique (OBL) astigmatism were less common, with minor variations among the groups.

**Table 2:** Distribution of astigmatism across atopic disease categories.

	Ocular atopic disease	Other atopic disorders	No atopic disease	Total	*p* value
Astigmatism power (n,%)					<0.001
<0.75 D	3939	83,037	685,462	772,438	
	83.8	84.9	86.2	86.0	
≥0.75, <3 D	694	13,923	104,083	118,700	
	14.8	14.2	13.1	13.2	
≥3 D	69	790	5814	6673	
	1.5	0.8	0.7	0.7	
Astigmatism axis (n,%)					<0.001
WTR	485	8679	64777	73941	
	10.3	8.9	8.1	8.2	
ATR	194	4342	32069	36605	
	4.1	4.4	4.0	4.1	
OBL	84	1692	13051	14827	
	1.8	1.7	1.6	1.7	

*D* dioptres, *WTR* with-the-rule, *ATR* against-the-rule, *OBL* oblique.

**Fig. 2:**
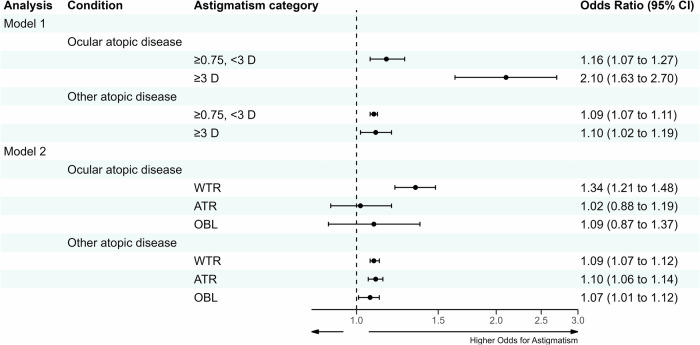
Adjusted multinomial regression analyses of the association between various atopic conditions and astigmatism. Models were adjusted for sex, country of birth, cognitive performance, socioeconomic status, and body mass index. Adolescents with ocular atopic disease (OAD) had increased odds for low-to-moderate and high astigmatism, as well as with-the-rule astigmatism. Other atopic disorders showed more modest point estimates. D dioptres, WTR with-the-rule, ATR against-the-rule, OBL oblique, CI confidence interval.

There was no interaction between gender and atopic disorders in relation to astigmatism (*p*_*interaction*_ = 0.652). Figure 2 depicts the results of the adjusted multinomial regression analyses. In general, point estimates in the unadjusted models were consistent with those in the adjusted models (Supplementary Table 2). Adolescents with OAD had increased ORs for low-to-moderate (OR 1.16, 95% CI 1.07–1.27) and high astigmatism (OR 2.10, 95% CI 1.63–2.70), along with an increased OR for WTR astigmatism (OR 1.34, 95% CI 1.21–1.48). In contrast, those with other atopic disorders showed more modest ORs for having low-to-moderate (OR 1.09, 95% CI 1.07–1.11) and high astigmatism (OR 1.10, 95% CI 1.02–1.19), with all axis orientations exhibiting statistically significant ORs close to 1.0.

### Sensitivity analysis

A dose-response relationship was observed in the prevalence of astigmatism with increasing severity of OAD (Supplementary Table 3A). Adolescents with mild OAD showed increased ORs for low-to-moderate astigmatism (OR 1.09, 95% CI 1.002–1.19) and a higher OR for high astigmatism (OR 2.03, 95% CI 1.57–2.64). In severe OAD, ORs accentuated and reached 1.60 (95% CI 1.22–2.12) for low-to-moderate astigmatism and 8.84 (95% CI 5.47–14.28) for high astigmatism (Supplementary Table 3B). There was also a dose-response relationship between the severity of OAD and ORs for WTR astigmatism, which increased from 1.22 (95% CI 1.09–1.35) in mild cases to 3.08 (95% CI 2.31–4.12) in severe cases (Supplementary Table 3C).

Stratification of the study population by myopia status demonstrated comparable results in the group without myopia, while results among adolescents with myopia attenuated (Supplementary Fig. 2).

Sub-analysis of 1331 adolescents newly diagnosed with OAD during military service revealed comparable findings (Supplementary Table 4A). The condition was associated with increased ORs for low-to-moderate astigmatism (OR 1.20, 95% CI 1.03–1.40) and high astigmatism (OR 1.62, 95% CI 0.96–2.75), although the latter was not statistically significant (Supplementary Table 4B). Furthermore, newly-diagnosed OAD was associated with increased odds for WTR and ATR astigmatism, with ORs of 1.27 (95% CI 1.05–1.52) and 1.31 (95% CI 1.03–1.67), respectively (Supplementary Table 4C).

## Discussion

The findings of this study shed light on the intricate relationship between atopic diseases, specifically OAD, and astigmatism among adolescents and young adults. Our results indicate an important association between OAD and an increased likelihood of both low-to-moderate (OR up to 1.60) and high astigmatism (OR up to 8.84), as well as a distinctive predominance of WTR astigmatism.

Our study’s cohort, comprising 897,811 adolescents, provides a substantial sample size, allowing for robust statistical analyses and a comprehensive exploration of the prevalence of astigmatism in the context of atopic diseases. The overall mean age of 17 years aligns with the age up to which most atopic conditions would have already manifested in the general population [2, 3, 19–21]. The prevalence of OAD in our cohort was on the lower side of previous reports (0.5% versus 0.003–7.3%) [2, 3], probably due to some amount of reporting bias, where only patients who were symptomatic during a short time preceding pre-conscription evaluation were assigned the corresponding FFS code. The prevalence of asthma, allergic rhinitis, atopic dermatitis, urticaria, and anaphylaxis was comparable to previous reports for the adolescent-young adult age group [19–23]. The male predominance and higher socioeconomic status of patients with atopic disease are also in concordance with previous reports [2, 3, 19–21], further validating our data.

Astigmatism is a common refractive error, which was detected in 14.0% of our cohort, in concordance with previous reports [9, 10]. Corneal astigmatism, the most common type of astigmatism in young people, [24] forms when the corneal shape deviates from sphericity, resulting in meridional variations in refractive power. Several factors have been implicated in astigmatism formation in young otherwise healthy eyes including eyelid pressure on the cornea, extraocular muscle tension, and genetic predisposition [24]. In OAD pressure may be exerted on the cornea through two separate mechanisms. First, the typical papillary reaction on the tarsal surface of the eyelids is located just above the upper and lower pole of the cornea, allowing for continuous direct pressure on this area. This hypothesis is further supported by the predominantly WTR astigmatic pattern found in patients with OAD in the current cohort. Another pressure-inducing mechanism related to OAD is eye rubbing [25]. Previous studies have demonstrated that eye rubbing induces corneal distortion, which may be transient or persistent, causing corneal astigmatism [5, 13–17]. This mechanism, however, does not explain the clear predisposition of patients with OAD for WTR astigmatism. Finally, inflammatory mediators play a significant role in OAD, arising from both the disease itself and the irritation caused by the recurrent eye rubbing [24, 26]. These inflammatory factors have been previously implicated in the pathophysiology of keratoconus [15, 26, 27]. While the role of inflammatory mediators in astigmatism formation is yet to be explored, their influence on corneal distortion in keratoconus raises the possibility that they may affect simple astigmatism as well.

The dose-response relationship observed in sensitivity analysis, where odds for astigmatism increased with the severity of OAD, adds to the strength of our findings. This aligns with previous studies that have proposed a progressive impact of mechanical trauma on corneal structure, leading to varying degrees of astigmatism [5, 26, 28, 29]. Moreover, our sub-analysis of young adults newly diagnosed with OAD during military service reaffirms the association with astigmatism. The ORs were slightly more modest in this subgroup, presumably due to the shorter duration of the effect of OAD in these patients.

While patients with OAD exhibited up to a 2.1-fold odds for astigmatism, other atopic conditions were associated with lower point estimates. This finding is in contrast to their significant implication as risk factors for keratoconus [1]. While the mechanism behind their association with keratoconus is unknown, it seems that simple astigmatism formation requires direct irritation and pressure on the ocular surface, both of which are not typically present in other atopic conditions apart from OAD.

Regardless of the mechanism by which astigmatism is associated with OAD, the existence of such an association emphasizes the importance of proper disease control, especially in children with moderate-severe OAD. High astigmatism has been shown to induce amblyopia formation in young children [30]. Furthermore, uncorrected astigmatism has been shown to decrease patients’ vision-related quality of life and productivity and pose a significant economic burden on patients and their families [11]. Thus, by achieving optimal control of OAD, not only can we alleviate patients’ discomfort and minimize disease-related symptoms and sequelae, but we may also reduce the economic burden associated with astigmatism formation in this population. Since OAD is a highly prevalent condition in children and young adults worldwide, it becomes imperative to address its potential impact on astigmatism and implement strategies for effective management.

This study has several limitations. Due to its retrospective nature and the FFS coding system, data regarding atopic disease severity, duration, and progression were limited. It was also impossible to separate between AKC and VKC when analysing the data. Evaluation of keratoconus severity was constrained by the lack of precise tomographic data, which may have also led to misinterpretation of some topographic images as non-keratoconus, particularly in the absence of posterior elevation data typically available in tomographic imaging. In addition, a causative effect of atopic disease may only be assumed based on previous research, but could not be directly established due to the study design. Future prospective studies with more detailed ophthalmological assessments, including corneal tomography and timely follow-up could provide additional insights into the temporal aspects of this association. Furthermore, the role of inflammation in the progression of keratoconus warrants exploration in future research. This could include tear film osmolarity and point-of-care matrix metalloproteinase testing, given the growing evidence linking inflammation to keratoconus progression and severity [31]. Lastly, additional studies are needed to establish the mechanisms by which astigmatism formation may be propagated by atopic disease, regardless of direct ocular involvement.

To conclude, the current study presents a cohort of nearly 900,000 adolescents and young adults who underwent systematic standard evaluation for ocular as well as systemic pathologies. A significant association was demonstrated between atopic conditions, particularly OAD, and astigmatism, which remained consistent after adjusting for possible confounders. These findings not only deepen our understanding of the pathogenesis of astigmatism but also hold implications for clinical practice and public health, emphasizing the importance of early detection and management of atopic conditions to mitigate potential visual sequelae.

## Summary

### What was known before

- Astigmatism significantly affects patients’ visual function - Ocular atopic disease is a highly prevalent disorder in childhood and adolescence.

### What this study adds

- Ocular atopic disease was demonstrated to be significantly related to higher prevalence of astigmatism.
- A dose-response relationship was observed between astigmatism and ocular atopic disease.

## Supplementary information

Supplementary Material

## Data Availability

The datasets generated during and/or analysed during the current study are not publicly available due to confidentiality restrictions but are available from the corresponding author on reasonable request.
