# Supplementary material for: Atopic disease and astigmatism: a population-based study
Source: Eye (Lond). 2025 Feb 28;39(9):1694–9. doi: 10.1038/s41433-025-03712-6 (PMC12130233; doi:10.1038/s41433-025-03712-6)
Supplement: Supplementary file 1 — Supplementary Material [file 41433_2025_3712_MOESM1_ESM.docx]

**Supplementary Material**

**Supplementary Table 1.** Distribution of astigmatism across atopic disorders.

**Supplementary Table 2.** Odds ratios for astigmatism in unadjusted models.

**Supplementary Table 3.** Odds ratios for astigmatism by ocular atopic disease severity.

**Supplementary Table 4.** Analysis of adolescents with a new diagnosis of ocular atopic disease during military service.

**Supplementary Figure 1.** Atopic diseases prevalence per 100,000 adolescents throughout the study years.

**Supplementary Figure 2.** Adjusted multinomial regression analyses of the association between various atopic conditions and astigmatism - stratification by myopia status.

**Supplementary Table 1.** Distribution of astigmatism across atopic disorders.

|  | | **Ocular atopic disease** | | **Asthma** | | **Allergic rhinitis** | | **Atopic dermatitis** | | **Angioedema**  **and urticaria** | | **Anaphylaxis** | |
| --- | --- | --- | --- | --- | --- | --- | --- | --- | --- | --- | --- | --- | --- |
|  | | With | Without | With | Without | With | Without | With | Without | With | Without | With | Without |
| **Astigmatism power** | **<0.75 D** | 3939 | 768499 | 43079 | 729359 | 46468 | 725970 | 10646 | 761792 | 1076 | 771362 | 5004 | 767434 |
|  |  | 83.8% | 86.0% | 85.0% | 86.1% | 85.3% | 86.1% | 84.7% | 86.1% | 83.9% | 86.0% | 85.1% | 86.0% |
|  | **≥0.75, <3 D** | 694 | 118006 | 7195 | 111505 | 7603 | 111097 | 1816 | 116884 | 190 | 118510 | 828 | 117872 |
|  |  | 14.8% | 13.2% | 14.2% | 13.2% | 13.9% | 13.2% | 14.5% | 13.2% | 14.8% | 13.2% | 14.1% | 13.2% |
|  | **≥ 3 D** | 69 | 6604 | 418 | 6255 | 432 | 6241 | 100 | 6573 | 16 | 6657 | 48 | 6625 |
|  |  | 1.5% | 0.7% | 0.8% | 0.7% | 0.8% | 0.7% | 0.8% | 0.7% | 1.2% | 0.7% | 0.8% | 0.7% |
| **Astigmatism axis** | **<0.75 D** | 3939 | 768499 | 43079 | 729359 | 46468 | 725970 | 10646 | 761792 | 1076 | 771362 | 5004 | 767434 |
|  |  | 83.8% | 86.0% | 85.0% | 86.1% | 85.3% | 86.1% | 84.7% | 86.1% | 83.9% | 86.0% | 85.1% | 86.0% |
|  | **ATR** | 194 | 36411 | 2279 | 34326 | 2431 | 34174 | 537 | 36068 | 49 | 36556 | 267 | 36338 |
|  |  | 4.1% | 4.1% | 4.5% | 4.1% | 4.5% | 4.1% | 4.3% | 4.1% | 3.8% | 4.1% | 4.5% | 4.1% |
|  | **WTR** | 485 | 73456 | 4433 | 69508 | 4709 | 69232 | 1166 | 72775 | 137 | 73804 | 513 | 73428 |
|  |  | 10.3% | 8.2% | 8.7% | 8.2% | 8.6% | 8.2% | 9.3% | 8.2% | 10.7% | 8.2% | 8.7% | 8.2% |
|  | **OBL** | 84 | 14743 | 901 | 13926 | 895 | 13932 | 213 | 14614 | 20 | 14807 | 96 | 14731 |
|  |  | 1.8% | 1.7% | 1.8% | 1.6% | 1.6% | 1.7% | 1.7% | 1.7% | 1.6% | 1.7% | 1.6% | 1.7% |
| **Total** | | 4702 | 893109 | 50692 | 847119 | 54503 | 843308 | 12562 | 885249 | 1282 | 896529 | 5880 | 891931 |
|  |  | 100.0% | 100.0% | 100.0% | 100.0% | 100.0% | 100.0% | 100.0% | 100.0% | 100.0% | 100.0% | 100.0% | 100.0% |

D- diopters; WTR, with-the-rule; ATR- against-the-rule; OBL-oblique.

**Supplementary Table 2A.** Odds ratios for astigmatism in an unadjusted model.

| **Condition** | | **Astigmatism power** | |
| --- | --- | --- | --- |
|  |  | **≥0.75, <3 D** | **≥3 D** |
| **Ocular atopic disease**  (n=4,702) | **OR (95% CI)** | 1.16 (1.07-1.26) | 2.07 (1.63-2.62) |
|  | ***p*-value** | <0.001 | 0.629 |
| **Other atopic disease** (n= 97,750) | **OR (95% CI)** | 1.10 (1.08-1.13) | 1.12 (1.04-1.21) |
|  | ***p*-value** | <0.001 | 0.003 |

D- diopters; OR- odds ratio.

**Supplementary Table 2B.** Odds ratios for astigmatism axis in an unadjusted model.

| **Condition** | | **Astigmatism axis** | | |
| --- | --- | --- | --- | --- |
|  |  | **WTR** | **ATR** | **OBL** |
| **Ocular atopic disease**  (n=4,702) | **OR (95% CI)** | 1.30 (1.19-1.43) | 1.05 (0.91-1.22) | 1.12 (0.90-1.39) |
|  | ***p*-value** | <0.001 | 0.486 | 0.305 |
| **Other atopic disease** (n= 97,750) | **OR (95% CI)** | 1.11 (1.08-1.13) | 1.12 (1.08-1.16) | 1.07 (1.02-1.13) |
|  | ***p*-value** | <0.001 | <0.001 | 0.009 |

D- diopters; WTR, with-the-rule; ATR- against-the-rule; OBL-oblique; OR- odds ratio; CI- confidence interval.

**Supplementary Table 3A.** Distribution of astigmatism by ocular atopic disease severity.

|  | **Mild ocular atopic disease** | **Severe ocular atopic disease** | **No ocular atopic disease** | **Total** | ***p*-value** |
| --- | --- | --- | --- | --- | --- |
| Astigmatism power (n,%) |  |  |  |  | <0.001 |
| <0.75 D | 3702 | 237 | 768499 | 772438 |  |
|  | 84.7% | 72.0% | 86.0% | 86.0% |  |
| ≥0.75, <3 D | 620 | 74 | 118006 | 118700 |  |
|  | 14.2% | 22.5% | 13.2% | 13.2% |  |
| ≥3 D | 51 | 18 | 6604 | 6673 |  |
|  | 1.2% | 5.5% | 0.7% | 0.7% |  |
| Astigmatism axis (n,%) |  |  |  |  | 0.075 |
| WTR | 421 | 64 | 73456 | 73941 |  |
|  | 62.7% | 69.6% | 58.9% | 59.0% |  |
| ATR | 175 | 19 | 36411 | 36605 |  |
|  | 26.1% | 20.7% | 29.2% | 29.2% |  |
| OBL | 75 | 9 | 14743 | 14827 |  |
|  | 11.2% | 9.8% | 11.8% | 11.8% |  |

D- diopters; WTR, with-the-rule; ATR- against-the-rule; OBL-oblique.

**Table 3B.** Odds ratios for astigmatism by ocular atopic disease severity.

| **Ocular atopic disease** | | | **Astigmatism power** | |  |
| --- | --- | --- | --- | --- | --- |
|  |  |  | **≥0.75, <3 D** | | **≥3 D** |
| **Mild form**  (n=329) | **OR (95% CI)** | 1.09 (1.002-1.19) | | 2.03 (1.57-2.64) |  |
|  | ***p*-value** | 0.046 | | <0.001 |  |
| **Severe form** (n=4,373) | **OR (95% CI)** | 1.60 (1.22-2.12) | | 8.84 (5.47-14.28) |  |
|  | ***p*-value** | 0.001 | | <0.001 |  |

D- diopters; OR- odds ratio; CI- confidence interval.

**Supplementary Table 3C.** Odds ratios for astigmatism axis by ocular atopic disease severity.

| **Ocular atopic disease** | | **Astigmatism axis** | | |
| --- | --- | --- | --- | --- |
|  |  | **WTR** | **ATR** | **OBL** |
| **Mild form**  (n=329) | **OR (95% CI)** | 1.22 (1.09-1.35) | 0.96 (0.82-1.13) | 1.03 (0.80-1.31) |
|  | ***p*-value** | <0.001 | 0.629 | 0.842 |
| **Severe form** (n=4,373) | **OR (95% CI)** | 3.08 (2.31-4.12) | 1.83 (1.14-2.93) | 1.98 (0.98-4.02) |
|  | ***p*-value** | <0.001 | 0.012 | 0.058 |

D- diopters; WTR, with-the-rule; ATR- against-the-rule; OBL-oblique; OR- odds ratio; CI- confidence interval.

**Supplementary Table 4A.** Distribution of astigmatism by ocular atopic disease – cases diagnosed during military service.

|  | **With ocular  atopic disease** | **Without ocular atopic disease** | **Total** | ***p*-value** |
| --- | --- | --- | --- | --- |
| Astigmatism power (n,%) |  |  |  | 0.018 |
| <0.75 D | 1185 | 767314 | 768499 |  |
|  | 83.7% | 86.1% | 86.0% |  |
| ≥0.75, <3 D | 215 | 117791 | 118006 |  |
|  | 15.2% | 13.2% | 13.2% |  |
| ≥3 D | 16 | 6588 | 6604 |  |
|  | 1.1% | 0.7% | 0.7% |  |
| Astigmatism axis (n,%) |  |  |  | 0.09 |
| WTR | 139 | 73317 | 73456 |  |
|  | 60.2% | 58.9% | 58.9% |  |
| ATR | 75 | 36336 | 36411 |  |
|  | 32.5% | 29.2% | 29.2% |  |
| OBL | 17 | 14726 | 14743 |  |
|  | 7.4% | 11.8% | 11.8% |  |

D- diopters; WTR, with-the-rule; ATR- against-the-rule; OBL-oblique.

**Supplementary Table 4B.** Analysis of adolescents with a new diagnosis of ocular atopic disease during military service – astigmatism power.

| **Condition** | | **Astigmatism power** | |
| --- | --- | --- | --- |
|  |  | **≥0.75, <3 D** | **≥3 D** |
| **Ocular atopic disease**  (n=1,331) | **OR (95% CI)** | 1.20 (1.03-1.40) | 1.62 (0.96-2.75) |
|  | ***p*-value** | 0.018 | 0.072 |

D- diopters; OR- odds ratio; CI- confidence interval.

**Supplementary Table 4B.** Analysis of adolescents with a new diagnosis of ocular atopic disease during military service – astigmatism axis.

| **Condition** | | **Astigmatism axis** | | |
| --- | --- | --- | --- | --- |
|  |  | **WTR** | **ATR** | **OBL** |
| **Ocular atopic disease**  (n=1,331) | **OR (95% CI)** | 1.27 (1.05-1.52) | 1.31 (1.03-1.67) | 0.78 (0.48-1.28) |
|  | ***p*-value** | 0.013 | 0.027 | 0.326 |

D- diopters; WTR, with-the-rule; ATR- against-the-rule; OBL-oblique; OR- odds ratio; CI- confidence interval.

**Supplementary Figure 1.** Atopic diseases prevalence per 100,000 adolescents throughout the study years.

**Supplementary Figure 2.** Adjusted multinomial regression analyses of the association between various atopic conditions and astigmatism - stratification by myopia status.


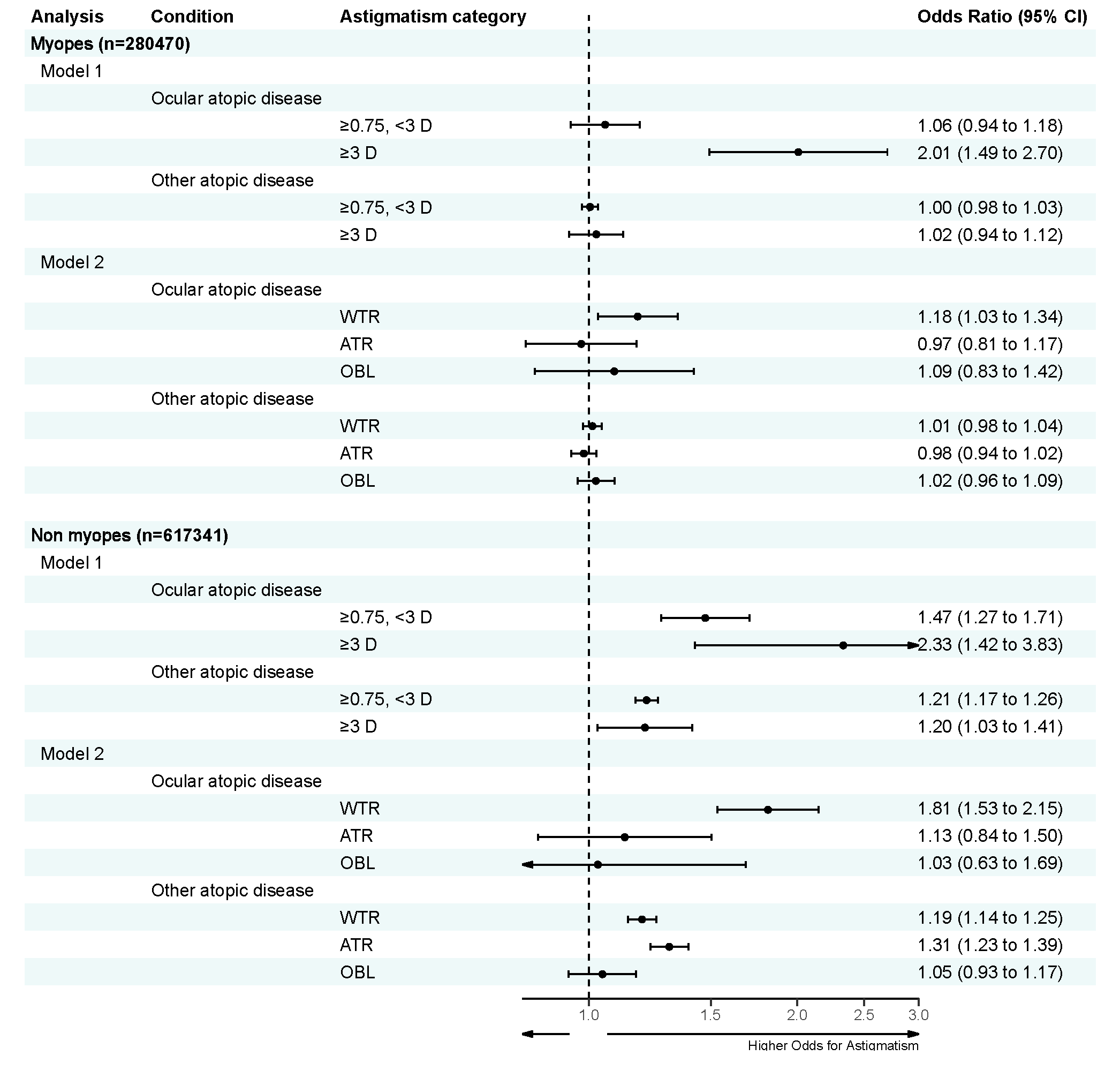


Models were adjusted for sex, country of birth, cognitive performance, socioeconomic status, and body mass index.
D- diopters; WTR- with-the-rule; ATR- against-the-rule; OBL- oblique; CI- confidence interval.
